# Progress in State Medicine

**Published:** 1903-05-23

**Authors:** 


*(To be continued.)*

### Progress in State Medicine.

### SMALL-POX.

Owing to the reappearance of small-pox from time to time in this country Sergeant[1] thinks it is of the first importance to have hospitals always in readiness for the admission of patients, and not to depend on hastily-built or adapted buildings. The Local Government Board require that the site of such hospitals must not have within a quarter of a mile of them either a hospital, whether for infectious diseases or not, or a workhouse, asylum, or any similar establishment, or a population of as many as 200 persons. Also the site must not have within half a mile of it a population of as many as 600 persons, whether in one or more institutions, or in dwelling houses. The hospital must not be used at one and the same time for the reception of cases of small-pox and any other class of disease. Sergeant points out that in forming a small-pox hospital district the size of the area and population have to be borne in mind; the larger the population within reasonable limits, the less proportionate number of beds will be required to be permanently provided, with, of course, a less expenditure involved. Consideration must be paid to the state of efficiency or otherwise of vaccination. The site should be as extensive as possible, and the construction of the building on the best principles. It is asserted in the Metropolitan Asylums Board Report[2] for 1901 that the errors in diagnosis of small-pox were unusually numerous. Out of 1,845 certified cases admitted to the South Wharf for shipment to Long Reach, no fewer than 237, or 12·7 per cent., were found at the wharf not to be suffering from small-pox; and in only eight cases more was the diagnosis corrected at the ships. The explanation is that medical men now certify cases which are only suspected, such as "contacts," who show some febrile disturbance, and who are notified before the eruption appears. The disease most frequently mistaken for small-pox was chicken-pox, of which 77 cases were sent to the wharf. Of acne there were 25 cases; of dermatitis, 18; syphilis, 17; urticaria, 14; eczema, 11; furunculus, 10; urticaria, vaccinia, and acute non-eruptive uninfectious diseases, 9 each; drug rashes and febricula, 6 each; impetigo and liche , 5 each; scabies, scarlet fever, and measles, 4 each; herpes, 3; and pemphigus, 2. Attention is called to the difficulty of distinguishing between the scars of primary vaccination and revaccination. The statements of patients are unreliable. Out of 82 cases, in 10 there was no cicatrix; in 27 only one or two scars were observed; in 26 the total scar area seen was under half square inch. The length of time said to have elapsed since revaccination was in 45 cases, over 20 years; in 24 cases between 20 and 10 years; in 12 cases between 10 and 4 years; and in one the interval was not ascertained. Of the 45 cases 9 died, of the 24, 3 died, and of the 12, 1 died. Of the hospital staff whose vaccination is under the charge of the medical superintendent, the medical and nursing staff, consisting of 98 persons, wardmaids and laundry-maids numbering 131, none took small-pox. Only one of the other employees, consisting of contractor's workmen and temporary workers, numbering 178, contracted small-pox. This was a butcher's driver who had not been re-vaccinated, who was admitted within the hospital precincts by mistake. The statistical tables show that of 1,282 vaccinated cases of all ages and degrees of vaccination, 127 died, or barely 10 per cent. Of 307 unvaccinated cases 119 died, or over 38 per cent. The earliest age at which a vaccinated child was attacked was between 3 and 4 years. It had unfoveated scars less than a third of a square inch in total area. The total number of vaccinated cases under 10 years old was 20, and all recovered. The earliest age at which a death of a vaccinated patient occurred was between 10 and 11 years. The next fatal cases among the vaccinated were between 15 and 20 years of age. The fatality rate of the vaccinated advances at later age-periods. Of 225 cases between 20 and 25 years there were

15 deaths, or 6·6 per cent.; 226 cases between 25 and 30 years, 17 deaths, or 7·5 per cent.; 170 cases between 30 and 35 years, 20 deaths, or 11·8 per cent.; 142 cases between 35 and 40 years, 26 deaths, or 18·3 per cent.; and 147 cases between 40 and 50 years of age, 34 deaths, or 20·3 per cent. After this age the fatality rate diminishes. On the other hand, among the unvaccinated the earlier years of life had many cases and many deaths—under 1 year 24 cases and 17 deaths, in the second year of life 31 and 10, in the fifth 20 cases and 6 deaths. In a total of 372 unvaccinated cases no fewer than 192, or over 51 per cent., were under 10 years old, while in a total of 1,282 vaccinated cases only 20, or less than 2 per cent., were under 10 years old. Among the unvaccinated, in a total of 119 deaths, no fewer than 65, or over 54 per cent., were in patients under 10 years old; whilst of the 127 deaths at all ages among vaccinated not a single death occurred in children under 10. Parkes[3] remarks that the recent outbreak of small-pox in London differs from its predecessors in that it had a short period, the maximum prevalence had been quickly attained, and the fall had been correspondingly rapid. In the first quarter of 1901 there was not a single death from small-pox in London, and there were only 7 cases; in the second quarter there were 7 cases and 2 deaths; in the third quarter 270 cases and 35 deaths. In the fourth quarter of 1901 the cases rose to 1,416 and the deaths to 192. In the first quarter of 1902 the cases were 4,470 and the deaths 734; the second quarter gave rise to 2,939 cases and 506 deaths; the third quarter to 356 cases and 73 deaths. He draws attention to the almost absolute freedom from small-pox enjoyed by London from the year 1885 to 1901, with the exception of the epidemic years of 1893–94. There was no such immunity from this disease in London in the inter-epidemic periods prior to 1885. This immunity dates from the removal of the London small-pox hospitals outside the metropolis to Long Reach, on the Thames. The total number of cases of small-pox notified in London during the year ending September 27th, 1902, was 9,171, with 1,505 deaths, giving a case-rate of 20 per 10,000 of the population, a death-rate of 0·33 per 1,000 and a fatality-rate of 16·4 per cent. of notified cases. The chief preventive measures employed were (1) revaccination of the general population, and of contacts with small-pox cases; (2) the removal of all notified cases to hospitals outside the Metropolitan boundaries. With regard to revaccination, he calculates that in Chelsea and Lambeth over 80 per cent. of the adult population have done nothing to protect themselves against the disease. The large majority of the people who decline to avail themselves of the protection of revaccination are the working classes. The only way to persuade this class to undergo revaccination is to offer them some monetary inducement. A small bonus at the time of the performance of the operation, and the promise to compensate for loss of wages in the event of disablement, is an argument which these people appreciate and not often reject. Local authorities have no power to expend public funds in this manner, but if a revaccinated population insures absence of small-pox, and money can effect the revaccination, even a very large expenditure might legitimately be incurred in this direction. If revaccination of all children at 13 or 14 years of age were compulsory, as it ought to be, extraordinary expenditure of this kind would after some years be very rarely necessary. Small-pox hospitals are undoubtedly exceedingly liable to act as centres of infection to the surrounding neighbourhoods. In any future building operations in the contiguity of occupied small-pox hospitals, revaccination of all the workmen engaged should be rigidly insisted upon, no matter what the extra cost may be in the price of the tender. The Government and the public must be impressed with the fact that recurring small-pox outbreaks are inevitable under the existing law as to vaccination; universal revaccination is the only protection against such outbreaks; and that to secure a fully protected community it is necessary (1) to make revaccination compulsory between the ages of 13 and 15; (2) to enable sanitary authorities to expend public funds in promoting revaccination.

[1] Public Health, Sept. 1902. [2] Brit. Med. Jour., Dec. 6, 1902.
[3] Public Health, Dec. 1902.

